# Advancing Antibody–Drug Conjugates: Precision Oncology Approaches for Breast and Pancreatic Cancers

**DOI:** 10.3390/cancers17111792

**Published:** 2025-05-27

**Authors:** Dhanvin R. Yajaman, Youngman Oh, Jose G. Trevino, J. Chuck Harrell

**Affiliations:** 1 Department of Pathology, Virginia Commonwealth University, Richmond, VA 23284, USA; yajamandr@vcu.edu (D.R.Y.); youngman.oh@vcuhealth.org (Y.O.); 2 Massey Comprehensive Cancer Center, Virginia Commonwealth University, Richmond, VA 23284, USA; jose.trevino@vcuhealth.org; 3 Department of Surgery, Virginia Commonwealth University, Richmond, VA 23284, USA

**Keywords:** antibody–drug conjugates (ADCs), triple-negative breast cancer (TNBC), pancreatic ductal adenocarcinoma (PDAC), cancer biomarkers, targeted cancer therapy, monoclonal antibodies, precision oncology

## Abstract

**Background/Objectives:** ADCs bring an innovative strategy to cancer treatment by conjugating powerful cytotoxic agents to the specificity of monoclonal antibodies. This review discusses recent advancements and challenges in the field of ADCs, along with future potential applications. **Methods:** Studies focused on the development of ADCs were reviewed. These include the effects of payload improvements, linker technologies, antibody engineering, and ADC internalization, which were particular topics of examination regarding their role in pancreatic ductal adenocarcinoma (PDAC) and triple-negative breast cancer (TNBC). The efficacy of some ADCs for pancreatic and breast cancers was compared. **Results:** In TNBC, ADCs such as sacituzumab govitecan and trastuzumab deruxtecan have improved progression-free survival in advanced cases. In contrast, PDAC ADC development is challenged by low antigen density and poor internalization; despite evidence of target engagement in early trials targeting mesothelin and MUC1, ADCs for PDAC have yet to achieve significant clinical efficacy or regulatory approval. **Conclusions:** While ADCs have significantly advanced treatment options in TNBC, PDAC remains a difficult target due to its stroma-rich microenvironment and lack of high-density, tumor-specific antigens. This article emphasizes the need for tailor-made ADC designs to enhance results in various types of cancers and provides valuable insight into future advancements in precision oncology.

## Simple Summary

An antibody–drug conjugate (ADC) is a cancer-inhibiting therapeutic agent that combines compounds, such as chemotherapy, with a targeted antibody. In recent years, the development and launch of new ADCs have been conducted for breast and pancreatic cancers. This article discusses ADC properties while comparing different targets, linkers, and payloads effectively used to treat these aggressive cancer types.

## 1. Introduction

### 1.1. Overview of Cancer

In 2020, an estimated 10 million people died from cancer globally, while nearly 600,000 cancer-related deaths were reported in the United States alone [1,2,3]. The lifetime probability of developing cancer is approximately 41.6% for men and 39.6% for women in the U.S., meaning nearly 4 in 10 men and women will be diagnosed with cancer during their lifetime [4]. According to the latest report, cancer is the second leading cause of death after heart disease in the nation, with almost 600,000 deaths [3]. Six of the top ten cancers have increasing incidence rates [4]. However, because of historical differences in preventative and equitable care across various populations, these statistics cannot be applied to the entire population [5,6]. For some cancers, the survival rates for patients of European, African, or Asian ancestry differ by more than two-fold. According to recent trends, over the next century, cancer may overtake cardiovascular disease as the leading cause of death in the majority of countries.

**Figure 1:**
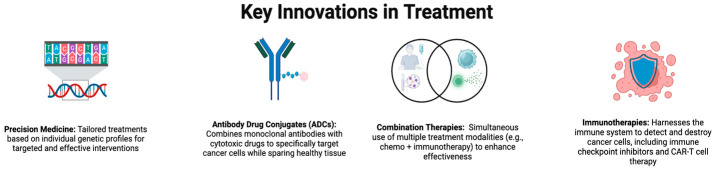
Key Innovations in Treatment. Created in BioRender. Y, D. (2025) https://BioRender.com/4ux4xk8.

Since the completion of the Human Genome Project, there have been major shifts in how cancers are diagnosed and treated. Drugs that target common mutations, novel antibodies, drug combination therapies, immunotherapies, and antibody–drug conjugates (ADCs) may yield more promising treatment (Figure 1). This review’s main focus is on the comparative study of antibody–drug conjugates currently being developed for pancreatic ductal adenocarcinoma (PDAC) and triple-negative breast cancer (TNBC). Both cancer types have limited therapeutic options. TNBC has seen notable clinical success with ADCs such as sacituzumab govitecan, while PDAC remains an area of unmet need due to its dense stroma, poor vascularization, and antigen heterogeneity. Comparing these two models offers insight into both the current potential and the limitations of ADC platforms due to both extremes in ADC clinical translation.

### 1.2. Pancreatic Cancer

The pancreas is the organ responsible for digestion and the control of blood sugar levels. Diagnosis of pancreatic cancer corresponds with a ~7 percent 5-year survival rate for patients in the US and has been rising in incidence and mortality rates [7,8]. In the early stages of pancreatic cancer, patients may present with only mild and non-specific symptoms. These include symptoms such as ascites, nausea, back pain, diarrhea, vomiting, steatorrhea, thrombophlebitis, jaundice, weight loss, hepatomegaly, anorexia, mass in the right upper quadrant of the body, abdominal pain, cachexia, epigastric pain, and dark urine [9].

Pancreatic cancer has multiple histological subtypes, each characterized by distinct clinical and molecular features. The most common subtype, PDAC, is responsible for nearly 80% of cases [10]. PDAC is characterized by aggressive progression, poor prognosis, and a high frequency of mutations in genes such as KRAS, TP53, CDKN2A, and SMAD4, which provide more than 50% mutational evidence in pancreatic cancer. Common mutations for these genes are shown in Table 1 [11,12,13,14,15].

Five percent of cases are defined as Pancreatic Neuroendocrine Tumors (PNETs), a less commonly occurring subtype that has less aggressive growth and a distinct treatment profile [10]. PNETs are treated with somatostatin analogs and targeted therapies and are frequently linked to genetic changes in MEN1, ATRX, and DAXX [16,17]. Mucinous cystic neoplasms and serous cystadenomas are representative of cystic pancreatic tumors, which in normal circumstances are pre-malignant and require different pathways of management, whereas other less common neoplasms, such as acinar cell carcinoma, possess unique clinical behaviors and molecular characteristics [18,19]. The incidence of different pancreatic cancer subtypes and the relevant mutations are shown in Table 2.

Hereditary factors are responsible for around 10% of cases of pancreatic cancer. Moreover, mutations in the genes responsible for Peutz-Jeghers syndrome, BRCA1/2, and Lynch syndrome are associated with the risk of developing pancreatic cancer [20]. The genetic findings have also facilitated the identification of molecular subtypes of PDAC, which include immunogenic, squamous, pancreatic progenitor, and aberrantly differentiated endocrine exocrine (ADEX) subtypes, with each presenting its own treatment strategies and prognostic implications [21,22].

Subtypes of pancreatic cancer differ significantly when it comes to prognosis and therapy. Chemotherapeutic agents have generally been used to treat PDAC, but its extremely high mutation load paves the way to experimental strategies such as ADCs and targeted therapies. In PNETs, peptide receptor radionuclide therapy and somatostatin analogs are the two primary forms of treatment, underscoring the need for individualized treatment plans [23].

Pancreatic cancer progresses through four stages; the earliest stage is when the tumor is confined within the pancreas, and it can be treated and/or resected. The survival rates for each tumor stage for PDAC and PNET are shown in Table 3. As the disease progresses, it spreads to the lymph nodes and ducts as well as surrounding organs. In the third stage, the tumor penetrates the main blood vessels, making it difficult to treat. The last stage of this disease is distant metastasis, in which cancer spreads to major organs of the body, like the liver. The survival rate at each stage is given below [24].

### 1.3. Breast Cancer

A total of 12% (one in eight) of women in the United States will be diagnosed with breast cancer in their lifetime. It is estimated that more than 2.3 million individuals are affected by this disease, with over 685,000 succumbing to it globally [25]. Breast cancer has a 5-year relative survival rate of over 80% in developed nations because of early detection and a plethora of treatment options. The rising rate of breast cancer in the United States has been offset by a drop in mortality due to therapeutic and technological advances in the treatment of breast cancer [26]. Breast cancer can be grouped into four different principal types based on the presence or absence of the estrogen receptor (ER), the progesterone receptor (PR), and HER2 (human epidermal growth factor 2-ERBB2) [27]. The four major subtypes are luminal A, luminal B, HER2-enriched, and basal-like/TNBC. Table 4 shows the characterizations of the different breast cancer subtypes as they relate to the presence or absence of receptors [28,29,30].

There are many risk factors associated with breast cancer. Women are 100 times more likely to develop breast cancer than men [31]. Furthermore, hereditary mutations in breast cancer gene 1 and breast cancer gene 2 (BRCA1 and BRCA2) may even increase the risk of breast cancer. In fact, BRCA1 and/or BRCA2 mutations account for 5 to 10% of all breast cancers and 20 to 25% of hereditary breast cancers. Additionally, age is a significant risk factor for breast cancer.

### 1.4. Antibodies and Cytotoxic Drugs in Cancer Therapy

Antibodies play a crucial role in the immune system, recognizing and binding to specific antigens on the surface of pathogens or abnormal cells [32]. Monoclonal antibodies offer relative specificity for tumor-associated antigens (TAAs); however, these antigens are often also expressed at low levels on healthy tissue, leading to potential on-target/off-tumor effects [33]. Thus, the actions of these antibodies involve several mechanisms, including immune-mediated destruction through complement-dependent cytotoxicity (CDC) or antibody-dependent cellular cytotoxicity (ADCC), immune checkpoint inhibition for enhancing an anti-tumor response from the body, and direct death of tumor cells by blocking essential growth signals [34]. The antibodies are very specific but often insufficient to destroy malignant tumors. Also, some cancers have developed resistance through decreased antigen expression or the utilization of other survival pathways [35].

However, conventional cytotoxic agents, such as chemotherapeutic agents, still occupy a prime place in cancer management because of their power to inhibit rapid cell division [36]. These drugs kill cancer cells through several means, such as alkylation of DNA, inhibition of nucleotide synthesis, topoisomerase interference, and alteration of microtubules. The only major disadvantage of these drugs is that they are not specific in action. Most of the patients suffer from severe side effects such as bone marrow suppression, gastrointestinal toxicity, and neuropathy due to these drugs because they affect non-cancer cells as well as their target cells [37,38,39]. Furthermore, many tumors would become resistant to chemotherapy by means of overcoming drug resistance through mechanisms such as enhanced drug efflux or more intense DNA repair, thereby limiting the long-term effectiveness of these treatments [40,41].

ADCs, the development of a new class of biopharmaceuticals, arose from the researchers’ aim of creating a more sophisticated method that would couple the potency of cytotoxic agents with the specificity of antibodies in view of the disadvantages of both cytotoxic drugs and the ordinary monoclonal antibodies. With the use of a specialized linker, a potent cytotoxic drug is chemically coupled to a monoclonal antibody in ADCs. Using the selective targeting of antibodies, ADCs deliver extremely toxic payloads to cancer cells, thereby minimizing off-target effects while maximizing therapeutic efficacy [42]. ADCs are transforming cancer therapy by eliminating the disadvantages of both conventional chemotherapy and antibody-based therapies and thus creating renewed hope for patients suffering from hard-to-treat cancers.

## 2. Antibody–Drug Conjugates

**Figure 2:**
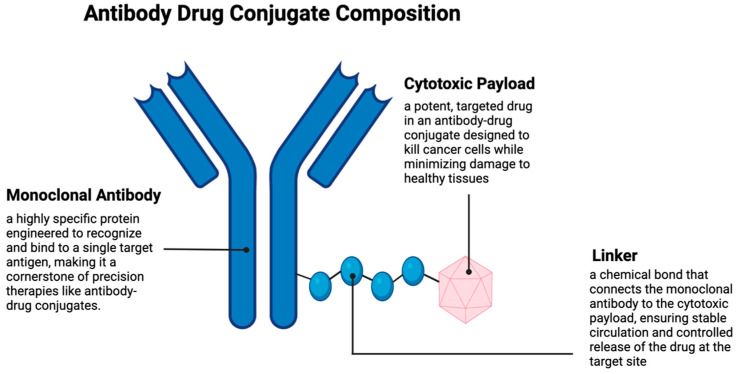
Antibody–drug conjugate composition. Created in BioRender. Y, D. (2025) https://BioRender.com/uapzr48.

ADCs are a new type of targeted therapy for cancer wherein monoclonal antibodies are attached to small-molecule cytotoxic drugs. The goal of an ADC is to deliver a toxic drug to malignant cells but spare healthy ones. ADCs represent an innovative strategy to target cancer therapy by fusing the toxicity of cytotoxic drugs with the specificity of mAbs. Because of this unique combination, ADCs have the precision to target and kill cancer cells while minimizing damage to healthy tissues, thereby lowering the toxicity and enhancing the therapeutic outcome. The three main components of ADCs include the cytotoxic payload, the linker, and the monoclonal antibody (Figure 2). Their balance of stability and effectiveness makes each an essential component of the therapeutic agent [43]. Compared to standard chemotherapy or immune checkpoint inhibitors, ADCs offer higher specificity and a favorable toxicity profile, especially in tumors with clear surface antigens. However, they may not be ideal for tumors with poor vascularization or low antigen density.

ADC efficacy is dependent on a multi-step process. The mAb component of the ADC first attaches itself to a very specific antigen, mostly proteins found on the surface of cancer cells. After binding, endocytosis allows the ADC to enter the cancer cell. Following internalization, lysosomal proteases cleave the linker (if cleavable), or the ADC is degraded entirely (if non-cleavable), releasing the cytotoxic payload within the cell. Finally, the released cytotoxic agent kills the targeted cancer cell by inhibiting critical functions such as microtubule formation and DNA replication [44]. In certain cases, the effect of a payload can spread beyond the target cell to neighboring cells, regardless of the expression of the antigen; this is called the bystander effect. This bystander effect arises when the released payload is membrane-permeable and can diffuse into adjacent cells, including those lacking target antigen expression [45]. This phenomenon becomes most prominent in tumors with heterogeneous antigen expression, where direct targeting cannot be relied upon to achieve complete tumor eradication. Various complications that affect the safety and efficacy of ADC therapy arise from the ability of the cytotoxic payload to diffuse into the microenvironment surrounding the tumor. The bystander effect is also an important element in the design and clinical use of ADCs since its magnitude depends on parameters such as payload properties, linker stability, and tumor architecture [46].

Selecting the appropriate target membrane protein is crucial for the efficacy and safety of ADCs.

The ideal target protein should have a few characteristics:

1. Be highly expressed on cancer cells with minimal presence in normal tissue, such as membrane proteins like HER2 (for breast cancer) and CD30 (for certain lymphomas), or TROP-2 (for breast/pancreatic cancer), which have demonstrated clinical success;
2. Be internalized efficiently after the ADC binds, ensuring the cytotoxic drug is delivered into the cancer cell [47].

The bystander effect has a major impact on treatment results when it comes to ADCs. An ADC is internalized and releases its cytotoxic payload when it attaches to its target antigen on a tumor cell. Combining the cytotoxic potential of conventional therapeutic agents with the specificity of monoclonal antibodies, ADCs represent a potent therapeutic approach to cancer therapy. Targeting cancer cells while leaving normal tissues relatively unscathed makes ADCs a promising approach for treating various cancers. The three component parts-linker, cytotoxic payloads, and antibodies-can be further optimized for greater safety and efficiency.

### 2.1. Antibodies

Most ADCs employ mAbs that confer antigen-specific targeting, allowing for selective delivery of cytotoxic agents to cancer cells overexpressing the target. mAbs serve to recognize and bind their respective antigens, which are either overexpressed or specifically expressed on the surface of malignant cells [48]. These antigens are proteins or receptors present in neoplastic tissues in much greater amounts than in healthy tissues. With the ADC targeting cancer cells with its cytotoxic payload, this selective binding will minimize off-target effects and enhance therapeutic selectivity [49]. The effectiveness of ADC therapy largely depends on high antigen expression on tumor cells and the antigen’s accessibility to antibody binding and internalization. Thus, careful selection becomes essential to the success of the ADC.

Upon binding of the mAb to the target antigen, the entire ADC complex is carried into the cancer cell by receptor-mediated endocytosis [50]. One of the key processes in delivering the cytotoxic payload and ensuring the trafficking of ADCs to lysosomes is internalization. The payload is released in the lysosomes at low pH and in an enzymatic environment to start the cytotoxic attack on the tumor cell [51]. Most of the payload is delivered to antigen-expressing malignant cells, although diffusion to surrounding cells—known as the bystander effect—can occur depending on payload permeability and linker design [52].

Some mAbs, apart from being used as the delivery mechanism, possess intrinsic immuno-modulating abilities that aid in triggering an overall therapeutic effect on ADCs. For instance, several mAbs activate their immune mechanisms of action, such as ADCC and CDC. ADCC refers to the process by which immune cells, such as NK cells, recognize target cells that are coated with antibodies and proceed to kill them through the release of cytotoxic molecules [53]. CDC is characterized by the activation of the complement system by antibodies and the consequent formation of a membrane attack complex, which destroys target cells [54]. In ADCC, Fc regions of the mAb engage Fcγ receptors on NK cells, triggering cytotoxic granule release [55]. Also, the CDC can activate the complement system to kill tumor cells. These supplementary mechanisms add synergistically to the therapeutic benefit of an ADC payload of direct cytotoxicity. Other antibody engineering developments aim to improve the efficacy and safety of mAbs used in ADCs. Technologies such as antibody humanization and affinity maturation are used to improve antigen-binding affinity while maintaining specificity; however, excessive affinity increases the risk of off-target interactions and must be carefully optimized. However, excessive affinity can result in slower dissociation and increased off-tumor binding. In fact, it can exacerbate off-tumor binding if not carefully controlled [56].

**Figure 3:**
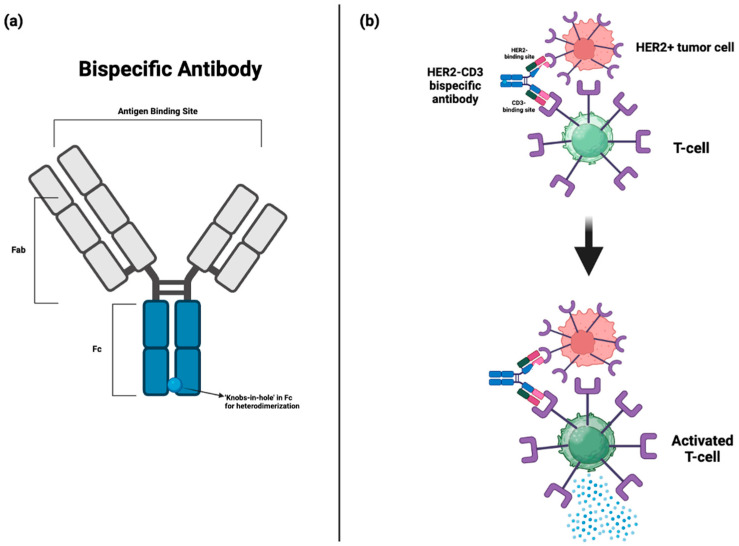
(**a**) Structural schematic of a bispecific antibody binding two antigens; (**b**) HER2-CD3 bispecific antibody engaging a tumor cell and T-cell, leading to T-cell-mediated cytotoxicity. Created in BioRender. Y, D. (2025) https://BioRender.com/35wheys.

Although not strictly ADCs, bispecific antibodies provide mechanistic insights relevant to antigen selection, immune modulation, and tumor heterogeneity—features that increasingly inform ADC design. Dual-targeting bispecific antibodies are engineered to simultaneously engage two distinct antigens, improving specificity or enhancing immune activation. These strategies inform the design of ADCs that must navigate antigen heterogeneity and immune evasion. Some designs may improve internalization, while others (e.g., T-cell engagers) function independently of internalization. While not ADCs, bispecific antibodies that target both tumor-associated antigens and CD3 on T cells—such as HER2/CD3 or TROP2/CD3—function by redirecting cytotoxic T cells toward tumor cells. These represent an alternative immunotherapeutic strategy distinct from the ADC platform. Bispecific and trispecific antibodies, designed today to bind with several targets in an integrated way, contribute to the advancement of detail and effectiveness in therapeutics and may reduce off-target effects by improving antigen specificity. The bispecific targeting tumor-associated antigens, such as HER2/CD3 against HER2-positive breast cancer or TROP2/CD3 against triple-negative breast cancer, would potentially activate the immune cells further by directing T cells against tumors instead, whereas trispecific designs would target various antigens from several tumor antigens simultaneously to counteract antigen escape, as in the case of HER2/EGFR/MUC1 for breast cancer or mesothelin/CA19-9/CD3 in pancreatic cancer (Figure 3) [57,58,59]. Structural innovations that modify the Fc region provide stabilizing effects and half-life extension. Heterodimerization technologies such as CrossMab and knobs-into-holes facilitate correct heavy/light chain assembly in bispecific antibodies, thereby improving manufacturability and structural fidelity [60,61]. For pancreatic cancer, which has such an immunosuppressive tumor microenvironment, bispecific antibodies with combinations of checkpoint blockade (PD-L1/CD3) or targeting macrophage reprogramming (CD47/SIRPα) are introduced to increase anti-tumor immunity, whereas trispecific constructs augment T cell persistence and infiltration through immune-stimulatory cytokines like IL-15 [62,63,64,65].

**Figure 4:**
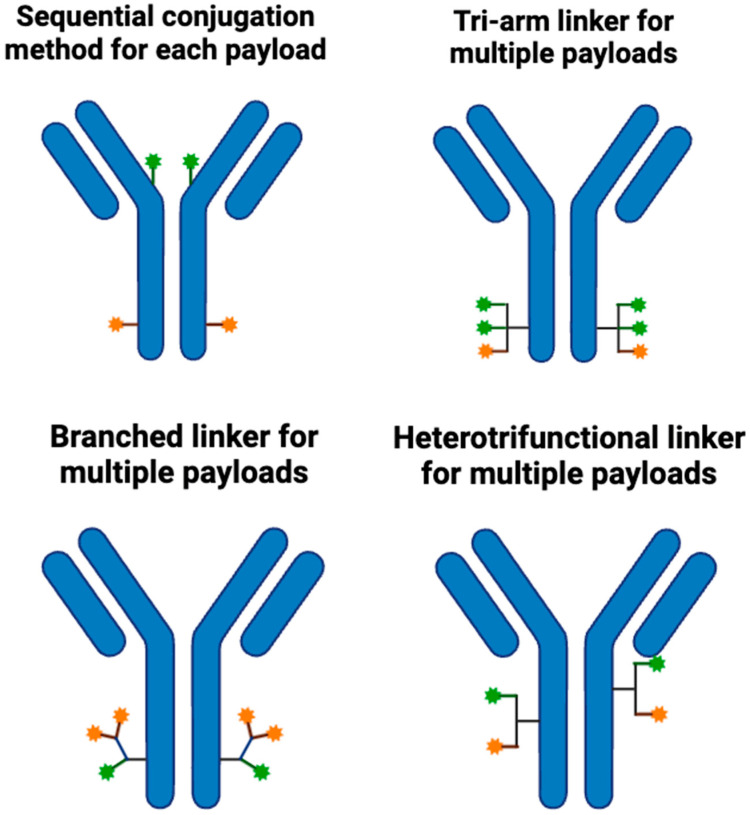
Linker conjugation possibilities. Created in BioRender. Y, D. (2025) https://BioRender.com/hnd6cxr.

The 2 + 1 (or 2:1) bispecific format—comprising two binding sites for a tumor antigen and one for an immune effector (e.g., CD3)—enhances avidity and tumor selectivity. This architecture has shown promise in overcoming resistance mechanisms in heterogeneous tumors, including breast and pancreatic cancers (Figure 4). The 2 + 1 bispecific antibody design is highly pertinent to breast and pancreatic cancers, not only because of potentially enhanced selectivity for tumors but also because of their influence on endosomal trafficking and antigen recycling, impacting the efficacy of the therapy at large [66]. Thus, bispecific antibodies targeting two HER2 epitopes in HER2-positive breast cancer can enhance HER2 clustering by facilitating the internalization of endosomes, coupled with inhibition of HER2 recycling back to the cell surface [67]. In tumors where receptor recycling plays a role in drug resistance, like with trastuzumab, this becomes especially valuable. Bispecifics may facilitate the degradation of HER2 in lysosomes instead of providing an avenue for recycling, thus augmenting therapeutic responses and diminishing the likelihood of escape mechanisms [68]. An example is pancreatic cancer, where mesothelin can be rapidly shed and recycled. An interesting feature of the mesothelin/CD3 2 + 1 bispecific is that it can increase retention of target antigen on the surface of the cell by controlling endosomal trafficking and, thus, enable T cell engagement and killing of tumor cells [69,70].

Glycosylation represents a very important posttranslational modification impacting the structure, stability, and functions of therapeutic antibodies, including ADCs [71]. Glycans attached to an ADC may influence its pharmacokinetics, immune interactions, and intracellular trafficking. Modifications in the Fc glycan structure, such as afucosylation, improve binding with Fcγ receptors, thus increasing the ADCC. Enhanced ADCC through Fc glycoengineering may provide additional therapeutic benefits, particularly in hematologic malignancies where immune effector mechanisms are more accessible [72]. In contrast, high-mannose glycoforms can increase the rate of clearance, and hence, it may be advantageous to minimize systemic toxicity. In addition, sialylation and bisected glycans can modify Fc receptor interactions and effectuate the fine-tuning of immune effector functions and ADC stability [73]. Given the natural heterogeneity of glycosylation in recombinant antibodies, glycoengineering is employed to optimize glycan structures, which can influence Fc receptor binding, antibody stability, and immune effector functions—thereby improving ADC efficacy and pharmacokinetics. In addition to modifying glycan structures, glycoengineering can involve glycosyltransferase knockouts or sialylation control. The most widely used method is enzymatic glycoengineering due to its precision and scalability in CHO cells.

This includes modifying glycan structure by different routes to increase ADC homogeneity, efficacy, and safety. Enzymatic glycoengineering, which utilizes glycosidases and glycosyltransferases, allows for the site-specific remodeling of glycans for the desired Fc properties [74]. Gene editing of Chinese hamster ovary (CHO) cell lines for the engineering of antibodies, widely used in producing ADCs via CRISPR/Cas9, allows for specific manipulation of the glycosylation pathway, thereby ensuring consistent glycan profiles. In addition, chemoenzymatic glycoengineering may allow for the incorporation of non-native glycans to facilitate site-specific drug conjugation for enhanced payload stability and controlled release [75]. Such techniques would improve ADC manufacturability and their therapeutic index and open the door to the next generation of glycoengineered ADCs capable of more efficacious action with considerably fewer off-target effects.

As antibody engineering continues to evolve, mAbs become increasingly sophisticated, securing their place as a key component in ADCs while extending their prospects in oncology and beyond.

**Figure 5:**
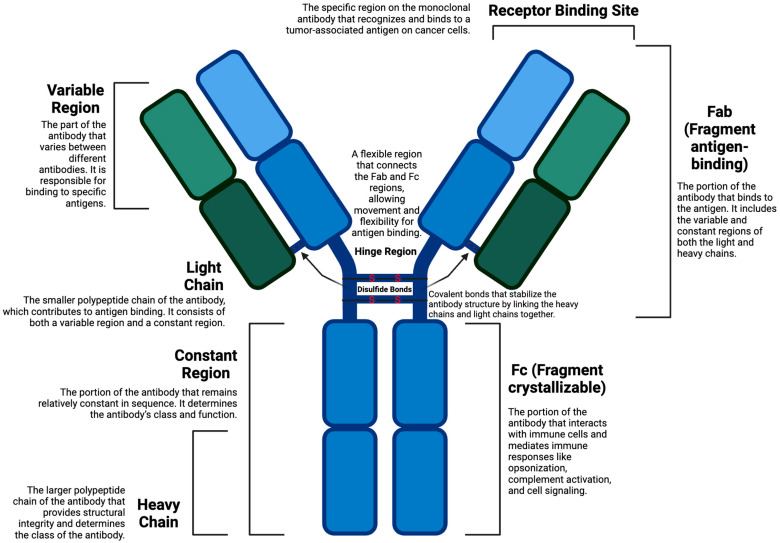
Detailed ADC Composition. Created in BioRender. Y, D. (2025) https://BioRender.com/xqcvyi3.

Host pharmacogenetics also plays a role in ADC tolerability and response. For instance, UGT1A1 polymorphisms can lead to increased toxicity in patients treated with SN-38-based ADCs, such as sacituzumab govitecan. Similarly, variability in Fcγ receptor expression may influence immune-mediated clearance of ADCs. These genetic factors must be considered in patient stratification for personalized ADC therapy. A detailed description of the composition of an ADC is shown in Figure 5.

### 2.2. Cytotoxic Payload

The cytotoxic payload is the functional unit of an ADC responsible for bringing about the death of a cancer cell [47]. Since ADC tumor delivery can be as low as 1–2% of the administered dose in some preclinical models, payload potency is critical to therapeutic success. Studies using site-specific conjugation, DAR-tuning (Drug-to-Antibody Ratio-tuning), and linker optimization have improved payload delivery. For example, trastuzumab deruxtecan achieves a DAR of ~8 with stable payload delivery; though associated with increased ILD risk, trastuzumab deruxtecan demonstrates superior tumoricidal activity compared to T-DM1, partially due to its higher DAR and cleavable linker design. The origin of the payload is mostly from highly potent chemotherapeutic agents, which in their free form would, therefore, be very toxic to use systemically. The concept behind such conjugation of drugs to monoclonal antibodies is to allow for direct targeting of the payload to the cancerous cells with minimal exposure to healthy tissues and systemic toxicity [52]. Payloads such as auristatins, maytansinoids, and calicheamicins destroy cells because they are intended for that purpose. They have been specifically selected because they act through important cellular processes. These agents are effective at picomolar concentrations, making them suitable for ADC delivery where only limited intracellular accumulation may occur [76].

The DAR, which refers to the average number of cytotoxic payload molecules conjugated per monoclonal antibody, is an essential parameter for influencing the efficacy of an ADC. This implies that DAR has a direct effect on the therapeutic index of an ADC since higher DAR means more potency but also possibly greater systemic toxicity and altered pharmacokinetics [77]. An optimized DAR should ideally provide enough payload to the tumor cell while ensuring antibody stability and minimizing off-target effects. In very low DAR ADCs, the amount of drug delivered may be insufficient to exert an anti-tumor effect, whereas very high DARs can promote aggregation, rapid clearance, and toxicity. Site-specific conjugation strategies have emerged to precisely control DAR and reduce batch heterogeneity [78].

The mechanism of action of the cytotoxic payload is critical to its efficacy. Most ADC payloads target essential cellular functions, such as DNA replication or microtubule dynamics [79]. Double-stranded breaks induced by DNA-damaging agents, such as calicheamicins, render the damage irreparable, leading to cellular death [80]. Microtubule inhibitors, such as MMAE and DM1, disrupt the mitotic spindle and stop cell division, bringing about apoptosis [81]. These mechanisms preferentially affect rapidly dividing cells, making them effective against proliferative tumors. However, the same potency of these agents demands stricter controls for releasing their payload to avoid non-specific toxicities.

The payload or cytotoxic agent is an indispensable component of ADCs. These agents potently kill the cancer cells if targeted appropriately. The two main classes of cytotoxic payloads used in ADCs are shown in Table 5 [82].

A critical challenge in ADC development is the selection and optimization of payloads, particularly between microtubule inhibitors (e.g., auristatins and maytansinoids) and DNA-damaging agents (e.g., calicheamicins and topoisomerase I inhibitors). While these payloads have demonstrated efficacy across multiple malignancies, their repeated use across different ADCs raises concerns regarding resistance mechanisms, cross-resistance, and the eventual exhaustion of viable treatment options for patients who develop resistance to a given ADC. One major pitfall of this convergence is that resistance to a single payload could render multiple ADCs ineffective. Resistance mechanisms may include alterations in drug efflux transporters such as ABCB1 (MDR1/P-gp), changes in intracellular trafficking that prevent lysosomal degradation and payload release, and upregulation of DNA repair pathways that mitigate the cytotoxic effects of DNA-damaging payloads [83]. For instance, patients treated with an ADC using MMAE may develop resistance through increased efflux activity, which could also reduce the efficacy of other MMAE-based ADCs [84]. Similarly, resistance to topoisomerase I inhibitors in one ADC may compromise the effectiveness of subsequent therapies using the same mechanism of action [85]. Another concern is the limited diversity of payload mechanisms. Most currently approved ADCs rely on either microtubule inhibition or DNA damage, leaving few alternative payload classes for patients who develop resistance [86]. Expanding payload diversity to include novel cytotoxic mechanisms, such as targeted protein degradation or immunostimulatory payloads, could mitigate this issue. Additionally, rational combination strategies involve pairing ADCs with agents that address known resistance mechanisms, such as immune checkpoint inhibitors, DNA repair inhibitors (e.g., PARP inhibitors), or agents that modulate TME to improve penetration. Additionally, rational combination strategies are increasingly guided by mechanistic insights into tumor biology and drug resistance. For instance, ADCs that induce immunogenic cell death can potentiate responses to immune checkpoint inhibitors by enhancing T-cell infiltration and activation. Similarly, DNA-damaging payloads such as topoisomerase I inhibitors create DNA lesions that synergize with PARP inhibitors in tumors harboring homologous recombination deficiencies. In desmoplastic or poorly vascularized tumors like PDAC, combining ADCs with agents that remodel the tumor microenvironment, such as hyaluronidase or TGF-β inhibitors, may improve drug penetration and payload delivery, thereby maximizing therapeutic benefit. When resistance to an ADC arises, treatment options become constrained, particularly if the patient’s tumor no longer responds to ADCs utilizing the same payload. In such cases, alternative strategies include switching to ADCs with different payloads, utilizing bispecific antibodies or chimeric antigen receptor T-cell therapies targeting the same antigen, or employing combination regimens that modulate resistance pathways. Additionally, emerging strategies such as epigenetic modulation and re-sensitization approaches are being explored to restore ADC efficacy in resistant cancers.

The continuous evolution of cytotoxic payloads has been driving the development of ADCs. Payload design innovations focus on improving specificity and potency while reducing the risk of resistance. Novel payloads with dual mechanisms of action have now been incorporated into some ADCs, enabling them to attack multiple vulnerabilities in cancer cells. Current ADC designs often employ site-specific conjugation strategies to achieve controlled DAR values, enhancing consistency and therapeutic efficacy. Additionally, research is being carried out on payloads that, beyond killing the cancer cells, would modulate the tumor microenvironment to inhibit metastasis or stimulate immune responses. These new developments emphasize how vital the payload is as a central component of ADCs, providing the therapeutic force that makes these conjugates such a powerful tool in precision oncology.

### 2.3. Linker

The use of linkers in ADCs is fundamental; linkers are the connections between mAbs and the cytotoxic payload, and the stability and efficacy of the therapy are considerably impacted by them [52]. The crucial task of the linker is to maintain stable conjugation of the payload covalently bound to the mAb during its circulation through the blood, to prevent premature drug release, and to minimize off-target toxicity [87]. The stable linker preserves the therapeutic window of the ADC, ensuring the payload is specifically given to the cancerous cells [79]. Therefore, the choice of linker design is critical to the overall efficacy and safety of the ADC.

The linkers may be broadly classified into cleavable and non-cleavable categories, and each uses a different mechanism for payload release. Cleavable linkers are created to elicit conditions faced specifically in the tumor microenvironment or that of the cell target [88]. Examples include pH-sensitive linkers, which release the payload in the acidic environment of lysosomes, while enzyme-sensitive linkers release the drug in response to protease activity. Common proteases, such as cathepsins, play a crucial role in activating enzyme-sensitive linkers by cleaving the antibody–drug conjugate, thereby releasing the cytotoxic payload within the lysosome. Yet another common variety is the disulfide-based linker, which releases the payload under reducing conditions in the cytoplasm [89]. It confers specificity from these unique release mechanisms, which means that maybe payload activation and exposure to normal tissues can be almost eliminated.

Non-cleavable linkers are not cleavable; this indicates their dependence on the degradation of the ADC-associated complex inside the target cell for the cytotoxic drug’s release [89]. When internalized, the ADC complex becomes degraded in lysosomes and releases the active form of the payload [90]. These linkers are mainly chosen because of their higher in vivo stability for resisting the premature release of any payload. Non-cleavable linkers are especially suitable to use when designing an ADC for targets low in accessibility to the extracellular enzymatic activities or where maximum control over the activation of the payload must be exerted [91]. Payloads may be modified in order to sustain activity following a lysosomal breakdown, so long as a non-cleavable linker is included.

Dual-cleavable linkers are structures used for maximum drug release with two areas of cleavage, usually one that cleaves by chemical means and the other by means of enzymatic action [92]. While dual-cleavable linkers are still largely experimental, they show promise in preclinical studies for enhancing drug release. These linkers not only enhance the stability of ADCs in circulation but also bring about rapid release of the payload within the tumor microenvironment or in cancer cells [93]. Common dual-cleavable linker strategies link acid-sensitive elements (hydrazone) or reduction-sensitive elements (disulfide) with protease-cleavable motifs (valine–citrulline) for the release of drugs from their carriers in a precise and controlled manner in the intracellular surroundings. This dual-trigger approach can improve ADC efficacy, limit premature systemic toxicity, and enhance the therapeutic index, thus being a promising strategy for optimization in targeted cancer therapy.

**Figure 6:**
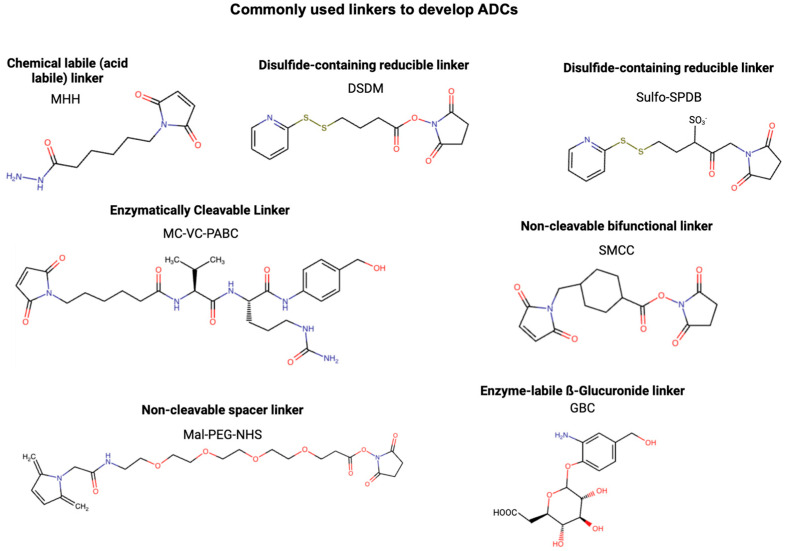
Chemical composition of commonly used linkers to develop ADCs. Created in BioRender. Y, D. (2025) https://BioRender.com/oszcmqk.

The type of linker affects other important features of the ADC, including pharmacokinetics (PK), pharmacodynamics (PD), and DAR. A well-designed linker would be equally stable and efficient in releasing payloads, taking into consideration the biophysical properties of the payload and the mAb. Linkers influence ADC PK by modulating systemic stability, drug release timing, and biodistribution. For instance, non-cleavable linkers enhance PK stability by preventing premature payload release in circulation but typically restrict bystander killing because the released payload is typically charged or hydrophilic and cannot diffuse across neighboring cell membranes. DAR impacts PK by altering clearance rates—higher DARs often lead to aggregation and faster clearance. They also influence PD by controlling the timing and location of payload release, directly impacting cytotoxic efficacy within tumor cells while minimizing off-target toxicity in healthy tissues. Precise linker design ensures that payloads are released intracellularly only after internalization, optimizing the therapeutic index. Linker technologies have moved past many of the previous limitations and have now enabled the incorporation of much more complex payloads, including those with dual mechanisms of action or immunomodulatory effects. New concepts, like cleavable linkers reactive to more than one stimulus or self-immolation linkers (linkers that degrade spontaneously after cleavage, enabling rapid and complete payload release), amplifying the effect of drug release, represent an extension of the scope of ADCs. These advances further develop linker technologies, which are continuously increasing the selectivity and potency of ADCs and solidifying them as one of the cornerstones of cancer therapy today. Figure 6 shows the linkers most commonly used to develop ADCs (developed using Marvin JS by Chemaxon, Budapest, Hungary).

### 2.4. Internalization of Antibodies

An important factor that influences the performance of ADCs is the degree of internalization of the antibodies, which dictates effective cytotoxic payload delivery into target cells. Several characteristics of the antibody may greatly influence its ability for internalization, starting with the target antigen. Antibodies that bind antigens associated with receptor-mediated endocytosis, such as growth factor receptors HER2 or EGFR, are more likely to promote internalization [94]. In contrast, antibodies receive considerably limited internalization triggered by antigens that do not have a specific endocytic route or are mostly shed into the extracellular milieu, thereby greatly hampering their therapeutic efficacy in ADCs [95].

The internalization efficiency of the antibody depends on the high-affinity and epitope-specific binding of the antibody. High-affinity antibodies ensure strong and continuous binding to the antigen of interest, leading to receptor clustering and increased chances for internalization [96]. Conversely, very high binding affinity may inhibit dissociation of the antibody from the receptor in endosomal compartments and thereby limit payload release. Secondly, the epitope targeted by the antibody can be another determinant that dictates its internalization fate. Antibodies targeting epitopes proximal to the cell membrane or within the domains of the receptor directly involved in signaling have a higher potential to induce internalization than those that target distal extracellular domains [97].

**Figure 7:**
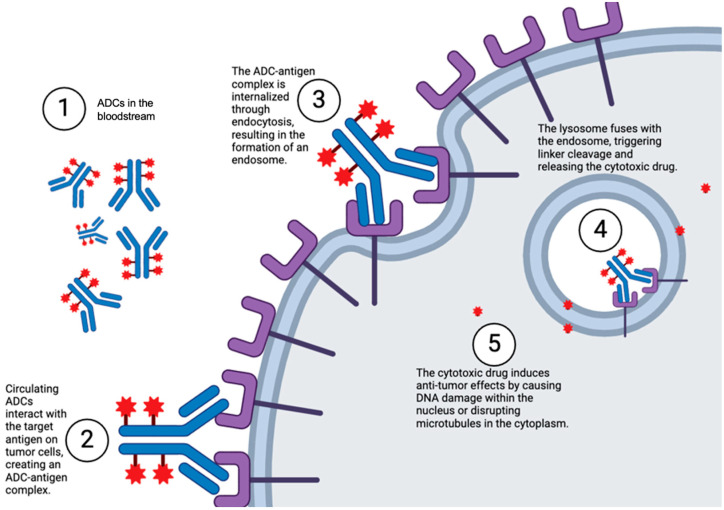
Internalization of Antibody–Drug Conjugates. Created in BioRender. Y, D. (2025) https://BioRender.com/4b7rj2u.

Internalization is more or less determined by other structural properties of the antibody, more specifically, the isotype and the Fc domain. While isotype generally affects effector function, engineered Fc domains can enhance uptake through FcγR-mediated mechanisms. The Fc domain may interact with Fc receptors on the cell surface to enhance endocytosis in certain instances [98]. Sometimes, such interactions will also enhance non-specific uptake by immune cells and lead to off-target effects. These interactions may inadvertently enhance off-target uptake by FcγR-expressing cells such as macrophages or dendritic cells. Most antibody engineering consists of modifications of the Fc region or of antibody fragments with the intention of optimizing internalization without compromising specificity [99]. These properties will increase successful internalization as well as overall efficacy in improving the chances of ADC success by engaging careful thought on these properties as well as designing the specific antibody (Figure 7).

## 3. Preclinical Studies and Lab Research

Preclinical studies of ADCs have been promising when testing models of pancreatic and breast cancer. In these cases, the researchers first identified several overexpressed proteins in cancer, including HER2 in breast cancer and MUC1 and CEA in pancreatic cancer. For instance, HER2 overexpression occurs in 15–20% of breast cancers [100]. Indeed, these target proteins have enabled ADCs to demonstrate some cytotoxic effects on cancer cell lines while leaving normal tissue unaffected, thus supporting the investigation into ADCs for these cancers. A major challenge in ADC therapy is overcoming tumor heterogeneity, where varying levels of antigen expression can impair uniform drug delivery; this property can also have a large impact on ADC efficacy, strengthening the ongoing research to improve target selection [101].

ADCs using HER2 (Human epidermal growth factor receptor 2) as a target are outlined in Table 6.

Other common targets for breast cancer include Trop-2, HER3, and FRα. The ADCs for these targets are in Table 7.

The other lesser-used targets for breast cancer are in Table 8, as well as their ADC information.

Proteins identified as targets for pancreatic cancer, as well as their respective antibody–drug conjugates and their mechanisms, are laid out in Table 9.

**Figure 8:**
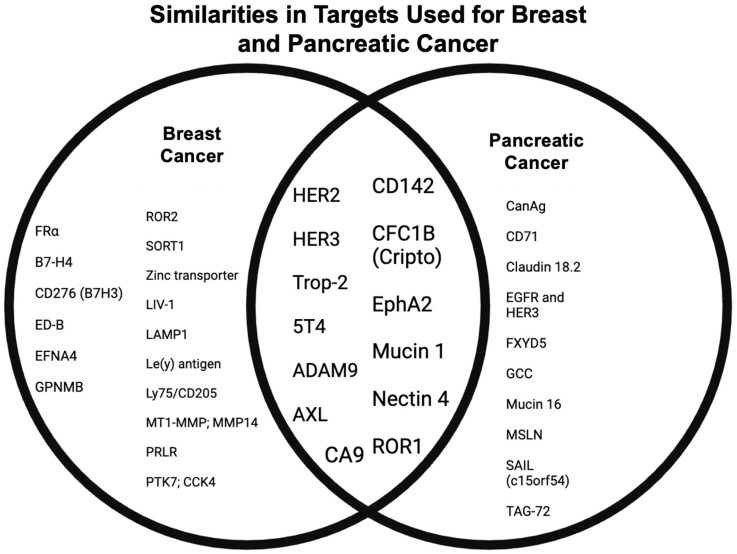
Similarities and differences in targets used in breast and pancreatic cancers. Created in BioRender. Y, D. (2025) https://BioRender.com/fqsxeal.

Similarities in targets used for breast and pancreatic cancer are highlighted in Figure 8.

## 4. Overview of Selected ADCs

ADCs have been studied as a potential avenue for cancer treatment since the 1980s. Early attempts at ADC development focused on targeted delivery by conjugating chemotherapeutic agents to antibodies. Gemtuzumab ozogamicin, the first-ever ADC approved for use in acute myeloid leukemia (AML), was approved in 2000. Following this, further developments of several ADCs for tumors like breast and pancreatic cancer were made [102].

For example, an essential regulatory milestone comprised the 2013 approval of T-DM1 (trastuzumab emtansine) for HER2-positive breast cancer [103]. Intended for new treatments to be offered to patients, ADCs have been translated into clinical practice thanks to expedited pathways provided by the FDA for Breakthrough Therapy Designation and Fast Track.

Breast cancer has been the focus of extensive ADC research, especially HER2-positive subtypes. With compelling proof of efficacy in clinical trials, trastuzumab emtansine (Kadcyla) and trastuzumab deruxtecan (Enhertu) have received approval for this indication in metastatic breast cancer [104]. ADCs are being tested in TNBC as well because of the gaps in clinical management of this aggressive subtype. Several ADCs have demonstrated strong efficacy in HER2-positive breast cancer, such as in HER2-positive cases. Some clinical trials with T-DM1 showed improved progression-free survival (PFS) and overall survival (OS) with regard to conventional therapies [105]. The safety profile indicates that some adverse events, such as fatigue, nausea, and thrombocytopenia, may be manageable; however, interstitial lung disease remains a concern with certain ADCs, notably trastuzumab deruxtecan [106].

Breast cancer ADC efficacy faces severe challenges due to the heterogeneity of the disease. Reliable biomarkers for patient selection, particularly in TNBC, remain a challenge. Future aspects will focus on increasing the tumor selectivity of ADCs, enhancing payload potency, and investigating ADC combinations with hormonal therapies and immunotherapy.

Pancreatic cancer is hard to treat because of its aggressive characteristics and often late-stage diagnosis. Patients are usually present with non-specific symptoms very early on in the course of their disease when the cancer is still localized. MUC1 and mesothelin are tumor markers with potential targeting for ADCs, which have shown good results in clinical studies. These early-phase clinical trials showed promise for anti-tumor activity, albeit slow in making headway in pancreatic cancers as a whole compared to other cancers.

In pancreatic cancer, some ADCs have shown moderate efficacy, whereas their safety profile remains under scrutiny. Certain common toxicities like neutropenia, thrombocytopenia, and rises in liver enzyme levels could possibly restrict the clinical applicability of some ADCs [43]. In pancreatic cancer, there is moderate efficacy in certain ADCs, while safety is still being assessed. Some common toxicities, such as neutropenia, thrombocytopenia, and elevations in liver enzyme levels, limit the clinical applications of some of these ADCs.

Challenges in ADC development for pancreatic cancer include the tumor microenvironment being very dense, which prevents adequate drug entry into the tumor. Other challenges include low tumor markers. Ongoing research efforts include better ADC delivery to the tumor site and improvement in payload toxicity profiling, as well as combination studies with immune checkpoint inhibitors and other targeted agents.

### 4.1. Comparison of Outcomes in Pancreatic vs. Breast Cancer

In breast cancer, ADCs have fared much better as a cancer treatment modality than in pancreatic cancer because of well-defined targets such as HER2. This inherent aggressiveness and desmoplastic nature of pancreatic tumors pose a significant challenge in delivering all PDAC therapies, ADCs included. Also, breast cancer trials come with well-established biomarker-driven methods, which provide them with a much higher success rate in clinical trials.

Lessons learned through breast cancer ADC development, such as target selection, combination strategies, and ADC design optimization, will drive future research endeavors for pancreatic cancer. The success seen in breast cancer strongly signals the need to develop innovative approaches for pancreatic cancer research, particularly in identifying better tumor-specific markers and more efficient delivery mechanisms.

### 4.2. Sequencing Antibody–Drug Conjugates (ADCs) in Cancer Therapy

ADCs have undoubtedly been a new frontier in cancer therapy as they combine the specificity of monoclonal antibodies with the therapeutic potential of chemotherapy agents. Still, over time, an ADC may end up being ineffective due to the development of resistance mechanisms or changes in tumor biology [79]. From a strategic standpoint, the sequencing of ADCs—replacing an ineffective ADC with another targeting a different payload or antibody epitope—can be a good way to bypass resistance in the hope of extending therapeutic gain [107].

The main reason for replacing an ADC is the development of resistance to its cytotoxic payload. Tumor cells can upregulate drug efflux pumps, such as P-glycoprotein, that decrease intracellular concentrations of the cytotoxic agent [108]. Mutations or adaptations to the target of the payload may also lead to a reduction in the efficacy of the drug. In such instances, switching to an ADC with a different payload or mechanism of action may restore efficacy, particularly when resistance is specific to the original cytotoxic agent or linker chemistry.

The other reason for replacing an ADC is a decrease in the antibody’s target antigen expression on tumor cells. Long exposure duration of ADC might exert selective pressures on the tumor cells, eventually leading to their downregulation or complete loss of the antigen, rendering the ADC incompetent in binding and delivering its payload [42]. In this case, the next possible step could be moving to an ADC for a different antigen still present in the tumor cells. Or, according to the heterogeneity of antigen expression in the tumor microenvironment, a bispecific or dual-targeting ADC may be considered [68].

Sequencing ADCs needs great care regarding the changing molecular profile of the tumor. Regular biopsies, liquid biopsies, or further biomarker assessments can signal the onset of resistance mechanisms or changes in antigen expression patterns to clinicians [109]. Oncologists can thereby increase the chance of an optimal outcome while delaying the progression of disease via personalized medicine applied to the unique biology of that patient’s cancer, individualized according to the resistance landscape of the tumor undergoing treatment.

## 5. Conclusions

ADCs have arisen as a highly promising field of innovation in the therapeutic arena for malignancy, combining the specificity of monoclonal antibodies with the cytotoxic effects of chemotherapeutic agents. By selectively killing cancer cells, ADCs have the potential to treat different types of malignancies with minimal systemic toxicity. This review discusses their impact on breast cancer, especially HER2-positive and triple-negative subtypes, benefiting from updated ADC design and implementation, leading to better outcomes for patients. Interestingly, pancreatic cancer presents special challenges in terms of both aggressiveness and tumor microenvironment complexity; at the same time, the advent of ADCs targeting specific biomarkers offers the promise of better treatment options. ADC successes differ between breast and pancreatic cancers, emphasizing the need to further advance the identification of new cancer targets, optimization of therapeutic payloads, and linker technology.

This review is limited by its narrative scope and may not capture all emerging preclinical ADC developments, particularly those outside PDAC and TNBC. Additionally, in vivo pharmacodynamics, manufacturing challenges, and cost considerations are not addressed in depth.

Similarities in learning from ADC’s progress in breast cancer will guide courses of action to address challenges faced in treating pancreatic cancer. Strategic refinement of ADC components, exploration of combinations with other therapies, and the ability to integrate biomarkers as part of clinical decision-making will be crucial to unlocking the potential of ADCs across diverse cancer types. With the ongoing clinical trial activity and research, ADCs form a key element within a future-focused precision oncology landscape that inspires new hope for patients facing tough diagnoses. The course metamorphoses from conception to a clinical application, well exemplifying the transformative reach intended for targeted therapies to fundamentally transform the treatment paradigm of cancer.

Future research should prioritize (1) the discovery of truly tumor-specific antigens, (2) novel payload classes beyond DNA-damaging agents and microtubule inhibitors, (3) more effective combinations with immunotherapy, and (4) the optimization of ADC penetration in dense stromal tumors like PDAC. More comprehensive pharmacogenetic stratification is also essential for precision-guided ADC deployment.

## Figures and Tables

**Table 1:** Common Genetic Mutations in Pancreatic Ductal Adenocarcinoma (PDAC).

Gene	Mutational Percentage	Location
SMAD4	19–50%	Chromosome #18 p21
CDKN2A	49–98%	Chromosome #9 p21
TP53	20–75%	Chromosome #17 p13.1
KRAS	70–95%	Chromosome #12 p12.1

**Table 2:** Incidence and Genetic Mutations of Pancreatic Cancer Subtypes.

Histological Subtype	Incidence (%)	Genetic Mutations
Pancreatic Ductal Adenocarcinoma	~80%	KRAS, TP53, CDKN2A, SMAD4
Pancreatic Neuroendocrine Tumors	~5%	MEN1, ATRX, DAXX
Acinar Cell Carcinoma	Rare	Unknown
Cystic Pancreatic Tumors	Rare	Pre-malignant markers

**Table 3:** Stage-Specific Survival Rates for Pancreatic Cancer Subtypes.

Tumor Stage	PDAC %	PNET %
Stage IA	14%	61%
Stage IB	12%	61%
Stage II	7%	52%
Stage III	3%	41%

**Table 4:** Breast Cancer Subtypes: Receptor Status, Incidence, Treatment, and Survival Rates.

ER	PR	HER2	Subtype	Incidence	Targeted Treatment	5, 10-Year Relapse-Free Survival
+	+	−	ER + luminal A	50–60%	Endocrine	95.6%, 89.5%
+	+	+/−	ER + luminal B	10–15%	Chemotherapy Endocrine + Anti-HER2	95.6%, 89.5%
−	−	+	HER2-Enriched	15–20%	Chemotherapy + Anti-HER2	92.9%, 92.9%
−	−	−	Basal-like/TNBC	15–20%	Chemotherapy	93.0%, 91.1%

**Table 5:** Classes of Cytotoxic Payloads Used in Antibody–Drug Conjugates (ADCs).

Category	Mechanism	Examples
Microtubule Inhibitors	These agents disrupt microtubule polymerization, essential structures for cell division.	Examples include MMAE and DM1, commonly used in ADCs like brentuximab vedotin and trastuzumab emtansine.
DNA Damaging Agents	These agents work by damaging the cancer cell DNA, preventing it from replicating.	Common examples include PBDs and calicheamicins, which cause DNA strand breaks leading to apoptosis.

**Table 6:** List of Antibody–Drug Conjugates (ADCs) Targeting HER2 and Their Linkers and Payloads.

ADC	Linker	Payload
AbGn-110	Proprietary linker	Biobetter cytotoxic payload
ADCT-502	A cathepsin B-cleavable valine–alanine linker	Tesirine, a clinically validated, potent pyrrolobenzodiazepine (PBD-based) dimer toxin (SG3249).
ARX788 HER2 ADC; ARX788	A non-natural amino acid linker para-acetyl-phenylalanine (pAcF)	Monomethyl Auristatin F (MMAF)
BAT8001	A novel uncleavable linker	A maytansine derivative
BB-1701	REsidue-SPEcific Conjugation	Eribulin
DHES0815A; RG6148	Disulfide linker	DNA minor groove crosslinking agent pyrrolo[2,1-c][1,4]benzodiazepine monoamide (PBD-MA)
Disitamab Vedotin; RC-48	A cleavable mc-val-cit-PABC-type linker.	Monomethyl Auristatin E (MMAE)
LCB14-0110; Herceptin-LC-LBG-MMAF	Proprietary linker	Monomethyl auristatin F (MMAF)
MEDI4276	Site-specific conjugation on mc-Lys-MMETA to 2 engineered cysteine residues on the heavy chain via a maleimidocaproyl linker.	MMETA, a Tubulysin Payload, also known as AZ13599185.
MI130004	Linker containing a maleimide group to enable conjugation to Cys residues.	PM050489
MM-302	PEG–DSPE	Liposomal doxorubicin
PF-06804103; Anti-NG-HER2 ADC	Cleavable valine–citrulline linker	Anti-Trop2 Aur0101
TAA013	Lysine–SMCC	DM1 (Maytansine)
Trastuzumab deruxtecan; DS-8201; DS-8201a; ENHERTU	A tetrapeptide linker, Gly–Phe–Leu–Gly (GFLG)	DXd
Trastuzumab duocarmazine; SYD985; Trastuzumab vc-seco-DUBA	A cleavable linker N-[2-(2 maleimidoethoxy)ethoxycarbonyl]-L-valyl-L-citrullinyl-p-aminobenzyloxycarbonyl-N-[2-(2-hydroxyethoxy)ethyl]-N-[2-(methylamino)ethyl]carbamoyl	Duocarmycin/Seco-DUBA
Trastuzumab Emtansine; T-DM1; Kadcyla	Noncleavable succinimidyl-4-(N-maleimidomethyl) cyclohexane-1-carboxylate (SMCC) linker	Maytansine DM1, a microtubule inhibitor.
Trastuzumab Imbotolimod; BDC-1001	A non-cleavable linker	A proprietary Toll-like receptor (TLR) 7/8 dual agonist
Trastuzumab rezetecan; SHR-A1811	A stable and cleavable linker.	SHR9265
Zanidatamab Zovodotin; ZW49	A proprietary cleavable, 1-maleimido-3,6,9-trioxadodecan-12-oyl-valyl-citrullyl linker	A novel, proprietary, N-acyl sulfonamide auristatin cytotoxin designed to take advantage of the enhanced antibody-HER2 internalization of ZW25.

**Table 7:** Antibody–Drug Conjugates (ADCs) Targeting Trop-2, HER3, and FRα in Breast Cancer.

ADC Target	ADC	Linker	Payload
FRα	BAT8006	Proprietary linker	A small molecule topoisomerase I inhibitor
	Farletuzumab Ecteribulin; MORAb-202	Cathepsin-cleavable linker	The microtubule-targeting agent (MTA), eribulin, a derivative of the macrocyclic polyether natural product halichondrin B.
	IMGN151	Stable, cleavable peptide linker	Maytansinoid derivative DM21
	Rinatabart Sesutecan; PRO1184; Rina-S	Proprietary linker	Exatecan payload
HER3	AMT-562	A via valine–alanine cleavable linker and a modified self-immolative PABC spacer (T800)	Site specifically conjugated to exatecan
	Patritumab Deruxtecan; U3-1402; HER3 ADC	Peptide cleavable linker (a tetrapeptide-based cleavable linker)	Deruxtecan, DX-8951 derivative (DXd, topoisomerase I inhibitor), a camptothecin derivative
Trop-2	BHV-1510	Proprietary linker	Proprietary potential best-in-class Topolx, site-specifically conjugated via Enzymatic (non-cysteine)
	RN927C	AcLys–VC–PABC; site-specific transglutaminase-mediated conjugation	A proprietary microtubule inhibitor (MTI) linker-payload, PF-06380101
	Sacituzumab Tirumotecan; SKB264; MK-2870	via site-specific conjugation and highly stable linker	A proprietary cytotoxic, belotecan-derived topoisomerase I inhibitor.

**Table 8:** Antibody–Drug Conjugates (ADCs) Targeting Lesser-Used Breast Cancer Targets.

ADC Target	ADC	Linker
5T4	ASN-004	Single-chain homo-dimer antibody, Fleximer linker technology; 3 Fleximer chains per antibody.
	PF 06263507; A1-mcMMAF; Anti-5T4 monoclonal antibody	A non-cleavable maleimidocaproyl linker
ADAM9	IMGC936	A stable tripeptide linker
AXL	Mecbotamab Vedotin; BA3011; CAB-Axl-ADC	A cleavable mc–val–cit–PABC-type linker on an average of 4 cysteinyl
	Mipasetamab Uzoptirine; ADCT-601	A cleavable (valine–alanine dipeptide as cathepsine B cleavage site) maleimide type linker
B7-H4	SGN-B7H4V	Protease-cleavable peptide linker, valine –citrulline
CA9	BAY79-4620; 3ee9	Valine–citrulline (vc)
CD142	Tisotumab Vedotin; Tivdak; HuMax-TF-ADC	A cleavable mc–val–cit–PABC type linker on an average of 3–4 cysteinyl
	XB002 ICON-2 Tissue Factor ADC	Protease cleavable valine–citrulline (vc) linker
CD276 (*B7H3*)	Mirzotamab Clezutoclax; ABBV-155; Mirzo-C	A cleavable dipeptide (valine–alanine) solubilizing linker.
CFC1B (Cripto)	BIIB-015	MCC
EphA2	MEDI-547	Stable linker maleimidocaproyl (mc)
ED-B	PYX-201	A cathepsin B-cleavable linker.
EFNA4	PF-06647263; anti-EFNA4-ADC	Hydrazone cleavable linker
GPNMB	Glembatumumab vedotin; CDX-011	A cleavable mc–val–cit–PABC-type linker
LAMP1	SAR428926	A disulfide-containing cleavable linker N-succinimidyl-4-(2-pyridyldithio)butyrate (SPDB)
Le(y) antigen	SGN-15; BMS-182248; BR96-DOX	A hydrazone linker
Ly75/CD205	MEN1309; OBT076	Cleavable linker
Mucin 1	SAR 566658	SPDB
MT1-MMP; MMP14	BT1718	A hindered disulfide linker
Nectin 4	Enfortumab Vedotin; Padcev; ASG-22ME; ASG-22MSE	A cleavable mc–val–cit–PABC-type linker
	Zelenectide Pevedotin (BT8009)	A valine–citrulline cleavable linker
PRLR	REGN2878-DM1; Anti-PRLR-ADC	Non-cleavable SMCC linker
PTK7; CCK4	Cofetuzumab Pelidotin; PF-06647020; ABBV-647	Cleavable valine–citrulline linker, a cleavable mc–val–cit–PABC-type linker
ROR1	Cirmtuzumab Vedotin; UC-961ADC3	Lysine-linker
	STRO-003	β-Glucuronidase-cleavable linkers
ROR2	Ozuriftamab Vedotin; BA3021; Anti-ROR2 ADC; CAB-ROR2-ADC	A cleavable mc–val–cit–PABC-type linker
SORT1	Sudocetaxel Zendusortide; TH1902	Cleavable linker
Zinc transporter LIV-1	Ladiratuzumab vedotin; SGN-LIV1A; Anti-LIV-1 ADC	A cleavable, mc–val–cit–PABC-type linker

**Table 9:** Antibody–Drug Conjugates (ADCs) Targeting Proteins in Pancreatic Cancer.

ADC Target	ADC	Linker	Payload
5T4	ZV05-ADC; 5T4-MMAF ADC; ZV05-mcMMAF; ZV0501	Proprietary linker	Monomethyl Auristatin F (MMAF)
ADAM9	Anti-ADAM9 ADC	Lysine-linked via a cleavable sulfo-SPDB linker	Maytansine-derived microtubule disruptor DM4
	Anti-ADAM9 ADC	Conjugated to engineered cysteine residues via a cleavable peptide linker	Indolinobenzodiazepine DNA-alkylating monoimine (DGN549)
	MGC028	bicyclononyne carbamoyl sulfamide Val–Ala–PABC	Exatecan, a topoisomerase I inhibitor payload
	IMGC936	A stable tripeptide linker.	DM21C
AXL	Mecbotamab Vedotin; BA3011; CAB-Axl-ADC	A cleavable mc–val–cit–PABC-type linker on an average of 4 cysteinyl	Monomethyl auristatin E (MMAE) (Vedotin)
CA9	BAY79-4620	Valine–citrulline (vc)	Monomethyl Auristatin E (MMAE)
CanAg	Cantuzumab mertansine; huC242-DM1; SB-408075	A stable thiopentanoate linker (or reducible SPP (N-succinimidyl 4-(2-pyridyldithio)) linker)	Maytansinoid antimicrotubule agent DM1|N(sup 2′)-deacetyl-N(sup 2′)-(3-sulfanylpropanoyl)maytansine
CD71	AbGn-107; Ab1-18Hr1	Cleavable linker	Tubulin inhibitor DM4
CD142	Tisotumab Vedotin; Tivdak; HuMax-TF-ADC	A cleavable mc–val–cit–PABC-type linker on an average of 3–4 cysteinyl	Microtubule disrupting agent monomethyl auristatin E (MMAE)
CFC1B	BIIB015	MCC	Maytansinoid derivative, DM4
Claudin 18.2	ATG-022	mc-vc-PABC-MMAE	Monomethyl auristatin E (MMAE)
	CMG901	A cleavable linker	
	EO-3021; SYSA1801	A cleavable linker	
	IMAB362-vcMMAE	Valine–citrulline linker	
	SOT102; SO-N102	Site Specific; non-cleavable Amide/Peptide Linker	PNU-159682, an anthracycline derivative.
	TQB2103	An enzymatically cleavable linker	A small-molecule toxin
EGFR and HER3	Izalontamab Brengitecan; BL-B01D1	A cathepsin B cleavable linker	A topoisomerase I inhibitor agent (Ed-04)
EphA2	MEDI-547	Stable linker maleimidocaproyl (mc)	Auristatin MMAF
FXYD5	EDC1; DYS-ADC	Proprietary linker	CEN-106
GCC	Indusatumab Vedotin; MLN-0264; TAK-264	A cleavable mc–val–cit–PABC-type linker.	Monomethyl auristatin E (MMAE)
HER2	ARX788 HER2 ADC	A non-natural amino acid linker para-acetyl-phenylalanine (pAcF)	Monomethyl Auristatin F (MMAF)
HER3	HER3-ADC	A cleavable valine–citrulline linker	Monomethyl auristatin E (MMAE)
Mucin 16	Sofituzumab vedotin; Anti-MUC16 ADC; RG7458; DMUC5754A	A cleavable mc–val–cit–PABC-type linker	Monomethyl auristatin E (MMAE)
MUC1	Clivatuzumab tetraxetan; hPAM4; hPAM4 IgG-DOTA	Conjugated, on an average of 4 to 7 lysyl, linked to the chelator by their N6.	Yttrium-90-labeled (90Y); Chelator tetraxetan (DOTA)
MSLN	DMOT4039A; RG7600	Protease-cleavable peptide linker.	Monomethyl auristatin E (MMAE)
Nectin 4	Enfortumab Vedotin; Padcev; ASG-22ME; ASG-22MSE	A cleavable mc–val–cit–PABC-type linker	Monomethyl Auristatin E (MMAE)
ROR1	Cirmtuzumab Vedotin; UC-961ADC3	Lysine linker	Monomethyl Auristatin E (MMAE)
SAIL c15orf54	IGN786	A maleimidocaproyl (mc) linker	Monomethyl auristatin F (MMAF)
TAG-72	Satumomab Penditide; OncoScint CR/OV	DTPA as a linker for the added In-111	Indium-111
Trop-2	RN927C	AcLys-VC-PABC	PF-06380101 (a Dolastatin 10 analogue)
	Sacituzumab Tirumotecan; SKB264; MK-2870	via site-specific conjugation and highly stable linker	A proprietary cytotoxic, belotecan-derived topoisomerase I inhibitor.
	Sacituzumab Govitecan	CL2A (pH-sensitive linker)	SN-38 (Topoisomerase I inhibitor, active metabolite of irinotecan)

## Abbreviations

The following abbreviations are used in this manuscript:

5T4	Oncofetal Antigen 5T4
ADC	Antibody–Drug Conjugate
ADCC	Antibody-Dependent Cellular Cytotoxicity
ADAM9	A Disintegrin and Metalloproteinase Domain 9
ADEX	Aberrantly Differentiated Endocrine Exocrine
AML	Acute Myeloid Leukemia
B7-H4	B7 Homolog 4
CA9	Carbonic Anhydrase IX
CanAg	Cancer Antigen
CEA	Carcinoembryonic Antigen
CD142	Cluster of Differentiation 142
CD276 (B7H3)	Cluster of Differentiation 276, B7 Homolog 3
CD71	Cluster of Differentiation 71
CDC	Complement-Dependent Cytotoxicity
CFC1B	Cripto, CFC1B
CHO	Chinese Hamster Ovary
DAR	Drug-to-Antibody Ratio
ED-B	Extra-Domain B
EFNA4	Ephrin A4
EGFR	Epidermal Growth Factor Receptor
ER	Estrogen Receptor
EphA2	Ephrin Type-A Receptor 2
FDA	Food and Drug Administration
FRα	Folate Receptor Alpha
FXYD5	FXYD Domain-Containing Ion Transport Regulator 5
GCC	Guanylate Cyclase C
GPNMB	Glycoprotein Non-Metastatic Melanoma Protein B
HER2	Human Epidermal Growth Factor Receptor 2
HER3	Human Epidermal Growth Factor Receptor 3
IL	Interleukin
ILD	Interstitial Lung Disease
LAMP1	Lysosomal-Associated Membrane Protein 1
Le(y) Antigen	Lewis Y Antigen
Ly75	Lymphocyte Antigen 75
mAb	Monoclonal Antibody
MUC1	Mucin 1
MSLN	Mesothelin
MT1-MMP; MMP14	Membrane-Type 1 Matrix Metalloproteinase
Nectin 4	Nectin Cell Adhesion Molecule 4
PDAC	Pancreatic Ductal Adenocarcinoma
PRLR	Prolactin Recepto
PTK7; CCK4	Protein Tyrosine Kinase 7, Cholecystokinin Tetrapeptide
ROR1	Receptor Tyrosine Kinase-Like Orphan Receptor 1
ROR2	Receptor Tyrosine Kinase-Like Orphan Receptor 2
SAIL	Secreted and Acidic Lysine-Rich Protein
SORT1	Sortilin
TAG-72	Tumor-Associated Glycoprotein 72
TNBC	Triple-Negative Breast Cancer
Trop-2	Trophoblast Antigen 2

## References

[1] Bray F., Laversanne M., Weiderpass E., Soerjomataram I. (2021). The ever-increasing importance of cancer as a leading cause of premature death worldwide. Cancer.

[2] Sung H., Ferlay J., Siegel R.L., Laversanne M., Soerjomataram I., Jemal A., Bray F. (2021). Global cancer statistics 2020: GLOBOCAN estimates of incidence and mortality worldwide for 36 cancers in 185 countries. CA Cancer J. Clin..

[3] Ahmad F.B., Anderson R.N. (2021). The leading causes of death in the US for 2020. JAMA.

[4] Siegel R.L., Giaquinto A.N., Jemal A. (2024). Cancer statistics, 2024. CA Cancer J. Clin..

[5] Zavala V.A., Bracci P.M., Carethers J.M., Carvajal-Carmona L., Coggins N.B., Cruz-Correa M.R., Davis M., de Smith A.J., Dutil J., Figueiredo J.C. (2021). Cancer health disparities in racial/ethnic minorities in the United States. Br. J. Cancer.

[6] Winn R., Winkfield K., Mitchell E. (2023). Addressing disparities in cancer care and incorporating precision medicine for minority populations. J. Natl. Med. Assoc..

[7] Eissa M.A., Lerner L., Abdelfatah E., Shankar N., Canner J.K., Hasan N.M., Yaghoobi V., Huang B., Kerner Z., Takaesu F. (2019). Promoter methylation of ADAMTS1 and BNC1 as potential biomarkers for early detection of pancreatic cancer in blood. Clin. Epigenet..

[8] Cai J., Chen H., Lu M., Zhang Y., Lu B., You L., Zhang T., Dai M., Zhao Y. (2021). Advances in the epidemiology of pancreatic cancer: Trends, risk factors, screening, and prognosis. Cancer Lett..

[9] Gheorghe G., Bungau S., Ilie M., Behl T., Vesa C.M., Brisc C., Bacalbasa N., Turi V., Costache R.S., Diaconu C.C. (2020). Early diagnosis of pancreatic cancer: The key for survival. Diagnostics.

[10] Mizrahi J.D., Surana R., Valle J.W., Shroff R.T. (2020). Pancreatic cancer. Lancet.

[11] Khan A.A., Liu X., Yan X., Tahir M., Ali S., Huang H. (2021). An overview of genetic mutations and epigenetic signatures in the course of pancreatic cancer progression. Cancer Metastasis Rev..

[12] Hwang R.F., Gordon E.M., Anderson W.F., Parekh D. (1998). Gene therapy for primary and metastatic pancreatic cancer with intraperitoneal retroviral vector bearing the wild-type p53 gene. Surgery.

[13] Schutte M., Hruban R.H., Geradts J., Maynard R., Hilgers W., Rabindran S.K., Moskaluk C.A., Hahn S.A., Schwarte-Waldhoff I., Schmiegel W. (1997). Abrogation of the Rb/p16 tumor-suppressive pathway in virtually all pancreatic carcinomas. Cancer Res..

[14] Blackford A., Serrano O.K., Wolfgang C.L., Parmigiani G., Jones S., Zhang X., Parsons D.W., Lin J.C.-H., Leary R.J., Eshleman J.R. (2009). SMAD4 gene mutations are associated with poor prognosis in pancreatic cancer. Clin. Cancer Res..

[15] Kim S.T., Lim D.H., Jang K.-T., Lim T., Lee J., Choi Y.-L., Jang H.-L., Yi J.H., Baek K.K., Park S.H. (2011). Impact of KRAS mutations on clinical outcomes in pancreatic cancer patients treated with first-line gemcitabine-based chemotherapy. Mol. Cancer Ther..

[16] Tirosh A., Kebebew E. (2020). Genetic and epigenetic alterations in pancreatic neuroendocrine tumors. J. Gastrointest. Oncol..

[17] Gujarathi R., Abou Azar S., Tobias J., Polite B.N., Setia N., Feinberg N., Appelbaum D.E., Keutgen X.M., Liao C.-Y. (2024). MEN1/DAXX/ATRX mutations enhance progression-free survival in gastroenteropancreatic neuroendocrine tumors treated with peptide receptor radionuclide therapy. Endocr.-Relat. Cancer.

[18] Testini M., Gurrado A., Lissidini G., Venezia P., Greco L., Piccinni G. (2010). Management of mucinous cystic neoplasms of the pancreas. World J. Gastroenterol..

[19] Thompson E.D., Wood L.D. (2020). Pancreatic neoplasms with acinar differentiation: A review of pathologic and molecular features. Arch. Pathol. Lab. Med..

[20] Das K.K., Early D. (2017). Pancreatic cancer screening. Curr. Treat. Options Gastroenterol..

[21] Gutiérrez M.L., Muñoz-Bellvís L., Orfao A. (2021). Genomic heterogeneity of pancreatic ductal adenocarcinoma and its clinical impact. Cancers.

[22] Connor A.A., Gallinger S. (2022). Pancreatic cancer evolution and heterogeneity: Integrating omics and clinical data. Nat. Rev. Cancer.

[23] Mavros M.N., Moris D., Karanicolas P.J., Katz M.H., O’Reilly E.M., Pawlik T.M. (2021). Clinical trials of systemic chemotherapy for resectable pancreatic cancer: A review. JAMA Surg..

[24] Rawla P., Sunkara T., Gaduputi V. (2019). Epidemiology of pancreatic cancer: Global trends, etiology and risk factors. World J. Oncol..

[25] Lei S., Zheng R., Zhang S., Wang S., Chen R., Sun K., Zeng H., Zhou J., Wei W. (2021). Global patterns of breast cancer incidence and mortality: A population-based cancer registry data analysis from 2000 to 2020. Cancer Commun..

[26] Sun Y.-S., Zhao Z., Yang Z.-N., Xu F., Lu H.-J., Zhu Z.-Y., Shi W., Jiang J., Yao P.-P., Zhu H.-P. (2017). Risk factors and preventions of breast cancer. Int. J. Biol. Sci..

[27] Waks A.G., Winer E.P. (2019). Breast cancer treatment: A review. JAMA.

[28] Cheng S.H.-C., Yu B.-L., Horng C.-F., Tsai S.Y., Chen C.-M., Chu N.-M., Tsou M.-H., Lin C.K., Shih L.-S., Liu M.-C. (2016). Long-term survival and stage I breast cancer subtypes. J. Cancer Res. Pract..

[29] Rej R.K., Roy J., Allu S.R. (2024). Therapies for the treatment of advanced/metastatic estrogen receptor-positive breast cancer: Current situation and future directions. Cancers.

[30] Iacopetta D., Ceramella J., Baldino N., Sinicropi M.S., Catalano A. (2023). Targeting breast cancer: An overlook on current strategies. Int. J. Mol. Sci..

[31] Siegel R.L., Miller K.D., Fuchs H.E., Jemal A. (2022). Cancer statistics, 2022. CA Cancer J. Clin..

[32] Marshall J.S., Warrington R., Watson W., Kim H.L. (2018). An introduction to immunology and immunopathology. Allergy Asthma Clin. Immunol..

[33] Farah R.A., Clinchy B., Herrera L., Vitetta E.S. (1998). The development of monoclonal antibodies for the therapy of cancer. Crit. Rev. ™ Eukaryot. Gene Expr..

[34] Augustyniak D., Majkowska-Skrobek G., Roszkowiak J., Dorotkiewicz-Jach A. (2017). Defensive and offensive cross-reactive antibodies elicited by pathogens: The good, the bad and the ugly. Curr. Med. Chem..

[35] Cassinelli G., Zuco V., Gatti L., Lanzi C., Zaffaroni N., Colombo D., Perego P. (2013). Targeting the Akt kinase to modulate survival, invasiveness and drug resistance of cancer cells. Curr. Med. Chem..

[36] Raghani N.R., Chorawala M.R., Mahadik M., Patel R.B., Prajapati B.G., Parekh P.S. (2024). Revolutionizing cancer treatment: Comprehensive insights into immunotherapeutic strategies. Med. Oncol..

[37] Nurgalieva Z., Liu C.-C., Du X.L. (2011). Chemotherapy use and risk of bone marrow suppression in a large population-based cohort of older women with breast and ovarian cancer. Med. Oncol..

[38] Akbarali H.I., Muchhala K.H., Jessup D.K., Cheatham S. (2022). Chemotherapy induced gastrointestinal toxicities. Adv. Cancer Res..

[39] Windebank A.J., Grisold W. (2008). Chemotherapy-induced neuropathy. J. Peripher. Nerv. Syst..

[40] Xue X., Liang X.-J. (2012). Overcoming drug efflux-based multidrug resistance in cancer with nanotechnology. Chin. J. Cancer.

[41] Salehan M., Morse H. (2013). DNA damage repair and tolerance: A role in chemotherapeutic drug resistance. Br. J. Biomed. Sci..

[42] Izzo D., Ascione L., Guidi L., Marsicano R.M., Koukoutzeli C., Trapani D., Curigliano G. (2025). Innovative payloads for ADCs in cancer treatment: Moving beyond the selective delivery of chemotherapy. Ther. Adv. Med. Oncol..

[43] Nguyen T.D., Bordeau B.M., Balthasar J.P. (2023). Mechanisms of ADC toxicity and strategies to increase ADC tolerability. Cancers.

[44] Pysz I., Jackson P.J., Thurston D.E. (2019). Introduction to antibody–drug conjugates (ADCs). https://aacrjournals.org/mct/article/3/6/661/234887/Inactivation-of-the-mitotic-checkpoint-as-a.

[45] Giugliano F., Corti C., Tarantino P., Michelini F., Curigliano G. (2022). Bystander effect of antibody–drug conjugates: Fact or fiction?. Curr. Oncol. Rep..

[46] Tang H., Liu Y., Yu Z., Sun M., Lin L., Liu W., Han Q., Wei M., Jin Y. (2019). The analysis of key factors related to ADCs structural design. Front. Pharmacol..

[47] Abdollahpour-Alitappeh M., Lotfinia M., Gharibi T., Mardaneh J., Farhadihosseinabadi B., Larki P., Faghfourian B., Sepehr K.S., Abbaszadeh-Goudarzi K., Abbaszadeh-Goudarzi G. (2019). Antibody–drug conjugates (ADCs) for cancer therapy: Strategies, challenges, and successes. J. Cell. Physiol..

[48] Adams G.P., Weiner L.M. (2005). Monoclonal antibody therapy of cancer. Nat. Biotechnol..

[49] Parit S., Manchare A., Gholap A.D., Mundhe P., Hatvate N., Rojekar S., Patravale V. (2024). Antibody-drug conjugates: A promising breakthrough in cancer therapy. Int. J. Pharm..

[50] Tashima T. (2022). Delivery of Drugs into Cancer Cells Using Antibody–Drug Conjugates Based on Receptor-Mediated Endocytosis and the Enhanced Permeability and Retention Effect. Antibodies.

[51] Ponziani S., Di Vittorio G., Pitari G., Cimini A.M., Ardini M., Gentile R., Iacobelli S., Sala G., Capone E., Flavell D.J. (2020). Antibody-drug conjugates: The new frontier of chemotherapy. Int. J. Mol. Sci..

[52] Drago J.Z., Modi S., Chandarlapaty S. (2021). Unlocking the potential of antibody–drug conjugates for cancer therapy. Nat. Rev. Clin. Oncol..

[53] Román V.R.G., Murray J.C., Weiner L.M. (2014). Antibody-dependent cellular cytotoxicity (ADCC). Antibody Fc.

[54] Gancz D., Fishelson Z. (2009). Cancer resistance to complement-dependent cytotoxicity (CDC): Problem-oriented research and development. Mol. Immunol..

[55] Ochoa M.C., Minute L., Rodriguez I., Garasa S., Perez-Ruiz E., Inogés S., Melero I., Berraondo P. (2017). Antibody-dependent cell cytotoxicity: Immunotherapy strategies enhancing effector NK cells. Immunol. Cell Biol..

[56] Marvin J.S., Lowman H.B. (2005). Antibody humanization and affinity maturation using phage display. Phage Display in Biotechnology and Drug Discovery.

[57] Kamakura D., Asano R., Yasunaga M. (2021). T cell bispecific antibodies: An antibody-based delivery system for inducing antitumor immunity. Pharmaceuticals.

[58] Liu J., Zhu J. (2024). Progresses of T-cell-engaging bispecific antibodies in treatment of solid tumors. Int. Immunopharmacol..

[59] Yao Y., Hu Y., Wang F. (2023). Trispecific antibodies for cancer immunotherapy. Immunology.

[60] Saxena A., Wu D. (2016). Advances in therapeutic Fc engineering–modulation of IgG-associated effector functions and serum half-life. Front. Immunol..

[61] Segués A., Huang S., Sijts A., Berraondo P., Zaiss D.M. (2022). Opportunities and challenges of bi-specific antibodies. Int. Rev. Cell Mol. Biol..

[62] Ye X., Yu Y., Zheng X., Ma H. (2024). Clinical immunotherapy in pancreatic cancer. Cancer Immunol. Immunother..

[63] Kiem D., Ocker M., Greil R., Neureiter D., Melchardt T. (2024). Enhancing anti-CD274 (PD-L1) targeting through combinatorial immunotherapy with bispecific antibodies and fusion proteins: From preclinical to phase II clinical trials. Expert Opin. Investig. Drugs.

[64] Lum L.G., Thakur A., Choi M., Deol A., Kondadasula V., Schalk D., Fields K., Dufrense M., Philip P., Dyson G. (2020). Clinical and immune responses to anti-CD3 x anti-EGFR bispecific antibody armed activated T cells (EGFR BATs) in pancreatic cancer patients. Oncoimmunology.

[65] Vahidi S., Touchaei A.Z., Samadani A.A. (2024). IL-15 as a key regulator in NK cell-mediated immunotherapy for cancer: From bench to bedside. Int. Immunopharmacol..

[66] Gu Y., Wang Z., Wang Y. (2024). Bispecific antibody drug conjugates: Making 1+ 1> 2. Acta Pharm. Sin. B.

[67] Zhang J., Ji D., Cai L., Yao H., Yan M., Wang X., Shen W., Du Y., Pang H., Lai X. (2022). First-in-human HER2-targeted bispecific antibody KN026 for the treatment of patients with HER2-positive metastatic breast cancer: Results from a phase I study. Clin. Cancer Res..

[68] Beishenaliev A., Loke Y.L., Goh S.J., Geo H.N., Mugila M., Misran M., Chung L.Y., Kiew L.V., Roffler S., Teo Y.Y. (2023). Bispecific antibodies for targeted delivery of anti-cancer therapeutic agents: A review. J. Control. Release.

[69] Zhang X., Yu Y., Peer C.J., Landsman R., Skorupan N., Cao L., Alewine C. (2022). Low serum mesothelin in pancreatic cancer patients results from retention of shed mesothelin in the tumor microenvironment. Transl. Oncol..

[70] Faust J.R., Hamill D., Kolb E.A., Gopalakrishnapillai A., Barwe S.P. (2022). Mesothelin: An immunotherapeutic target beyond solid tumors. Cancers.

[71] Duivelshof B.L., Jiskoot W., Beck A., Veuthey J.-L., Guillarme D., D’Atri V. (2019). Glycosylation of biosimilars: Recent advances in analytical characterization and clinical implications. Anal. Chim. Acta.

[72] Reusch D., Tejada M.L. (2015). Fc glycans of therapeutic antibodies as critical quality attributes. Glycobiology.

[73] Hayes J.M., Cosgrave E.F., Struwe W.B., Wormald M., Davey G.P., Jefferis R., Rudd P.M. (2014). Glycosylation and Fc receptors. Fc Receptors.

[74] Wang L.-X., Tong X., Li C., Giddens J.P., Li T. (2019). Glycoengineering of antibodies for modulating functions. Annu. Rev. Biochem..

[75] Ivanova A., Falcioni F. (2022). Challenges and opportunities for the large-scale chemoenzymatic glycoengineering of therapeutic N-glycosylated monoclonal antibodies. Front. Catal..

[76] Koehn F.E. (2012). Natural product cytotoxins as payloads for antibody drug conjugates. Natural Products and Cancer Drug Discovery.

[77] Lin K., Tibbitts J. (2012). Pharmacokinetic considerations for antibody drug conjugates. Pharm. Res..

[78] Kostova V., Désos P., Starck J.-B., Kotschy A. (2021). The chemistry behind ADCs. Pharmaceuticals.

[79] Yaghoubi S., Karimi M.H., Lotfinia M., Gharibi T., Mahi-Birjand M., Kavi E., Hosseini F., Sineh Sepehr K., Khatami M., Bagheri N. (2020). Potential drugs used in the antibody–drug conjugate (ADC) architecture for cancer therapy. J. Cell. Physiol..

[80] Weber G.F., Weber G.F. (2015). DNA damaging drugs. Mol. Ther. Cancer.

[81] Wang X., Gigant B., Zheng X., Chen Q. (2023). Microtubule-targeting agents for cancer treatment: Seven binding sites and three strategies. MedComm–Oncol..

[82] Fu Y., Ho M. (2018). DNA damaging agent-based antibody-drug conjugates for cancer therapy. Antib. Ther..

[83] Gandullo-Sánchez L., Ocaña A., Pandiella A. (2021). Generation of antibody-drug conjugate resistant models. Cancers.

[84] Yu S.-F., Zheng B., Go M., Lau J., Spencer S., Raab H., Soriano R., Jhunjhunwala S., Cohen R., Caruso M. (2015). A novel anti-CD22 anthracycline-based antibody–drug conjugate (ADC) that overcomes resistance to auristatin-based ADCs. Clin. Cancer Res..

[85] Sharma N.K., Bahot A., Sekar G., Bansode M., Khunteta K., Sonar P.V., Hebale A., Salokhe V., Sinha B.K. (2024). Understanding cancer’s defense against topoisomerase-active drugs: A comprehensive review. Cancers.

[86] Wang Z., Li H., Gou L., Li W., Wang Y. (2023). Antibody–drug conjugates: Recent advances in payloads. Acta Pharm. Sin. B.

[87] Qian L., Lin X., Gao X., Khan R.U., Liao J.-Y., Du S., Ge J., Zeng S., Yao S.Q. (2023). The dawn of a new era: Targeting the “undruggables” with antibody-based therapeutics. Chem. Rev..

[88] Xue Y., Bai H., Peng B., Fang B., Baell J., Li L., Huang W., Voelcker N.H. (2021). Stimulus-cleavable chemistry in the field of controlled drug delivery. Chem. Soc. Rev..

[89] Sheyi R., de la Torre B.G., Albericio F. (2022). Linkers: An assurance for controlled delivery of antibody-drug conjugate. Pharmaceutics.

[90] Leyton J.V. (2023). The endosomal-lysosomal system in ADC design and cancer therapy. Expert Opin. Biol. Ther..

[91] Fu Z., Li S., Han S., Shi C., Zhang Y. (2022). Antibody drug conjugate: The “biological missile” for targeted cancer therapy. Signal Transduct. Target. Ther..

[92] Bargh J. (2021). The Development of Sulfatase-Cleavable Linkers for Antibody-Drug Conjugates. Ph.D. Thesis.

[93] Ashman N., Bargh J.D., Spring D.R. (2022). Non-internalising antibody–drug conjugates. Chem. Soc. Rev..

[94] Slastnikova T.A., Ulasov A., Rosenkranz A., Sobolev A. (2018). Targeted intracellular delivery of antibodies: The state of the art. Front. Pharmacol..

[95] Chalouni C., Doll S. (2018). Fate of antibody-drug conjugates in cancer cells. J. Exp. Clin. Cancer Res..

[96] Yang C., He B., Zhang H., Wang X., Zhang Q., Dai W. (2023). IgG Fc affinity ligands and their applications in antibody-involved drug delivery: A brief review. Pharmaceutics.

[97] Brandsma A.M., Jacobino S.R., Meyer S., ten Broeke T., Leusen J.H. (2015). Fc receptor inside-out signaling and possible impact on antibody therapy. Immunol. Rev..

[98] Koenderman L. (2019). Inside-out control of Fc-receptors. Front. Immunol..

[99] Presta L.G. (2006). Engineering of therapeutic antibodies to minimize immunogenicity and optimize function. Adv. Drug Deliv. Rev..

[100] Baselga J. (2010). Treatment of HER2-overexpressing breast cancer. Ann. Oncol..

[101] Damelin M., Zhong W., Myers J., Sapra P. (2015). Evolving strategies for target selection for antibody-drug conjugates. Pharm. Res..

[102] Sassoon I., Blanc V. (2013). Antibody–drug conjugate (ADC) clinical pipeline: A review. Antibody-Drug Conjugates.

[103] Barok M., Joensuu H., Isola J. (2014). Trastuzumab emtansine: Mechanisms of action and drug resistance. Breast Cancer Res..

[104] Hurvitz S.A., Hegg R., Chung W.-P., Im S.-A., Jacot W., Ganju V., Chiu J.W.Y., Xu B., Hamilton E., Madhusudan S. (2023). Trastuzumab deruxtecan versus trastuzumab emtansine in patients with HER2-positive metastatic breast cancer: Updated results from DESTINY-Breast03, a randomised, open-label, phase 3 trial. Lancet.

[105] Liu F., Li Y., Yang D., Tang L., Yang Q., Jiang M., Tian L., An J. (2024). Meta-analysis of the clinical efficacy and safety of T-DM1 in the treatment of HER2-positive breast cancer. Indian J. Cancer.

[106] Chiu J.W.Y., Lee S.C., Ho J.C.-m., Park Y.H., Chao T.-C., Kim S.-B., Lim E., Lin C.-H., Loi S., Low S.Y. (2023). Clinical Guidance on the monitoring and management of Trastuzumab Deruxtecan (T-DXd)-Related adverse events: Insights from an Asia-Pacific Multidisciplinary Panel. Drug Saf..

[107] Sasso J.M., Tenchov R., Bird R., Iyer K.A., Ralhan K., Rodriguez Y., Zhou Q.A. (2023). The evolving landscape of antibody–drug conjugates: In depth analysis of recent research progress. Bioconjug. Chem..

[108] Breier A., Barancík M., Sulová Z., Uhrík B. (2005). P-glycoprotein-implications of metabolism of neoplastic cells and cancer therapy. Curr. Cancer Drug Targets.

[109] Pasini L., Ulivi P. (2019). Liquid biopsy for the detection of resistance mechanisms in NSCLC: Comparison of different blood biomarkers. J. Clin. Med..

